# Interannual and decadal variability of the North Equatorial Undercurrents in an eddy-resolving ocean model

**DOI:** 10.1038/s41598-018-35469-2

**Published:** 2018-11-20

**Authors:** Yiwen Li, Hailong Liu, Pengfei Lin

**Affiliations:** 1 State Key Laboratory of Numerical Modeling for Atmospheric Sciences and Geophysical Fluid Dynamics, Institute of Atmospheric Physics, Chinese Academy of Sciences, Beijing, 100029 China; 2 College of Earth Sciences, University of Chinese Academy of Sciences, Beijing, 100049 China

## Abstract

In the present study, the mean states of the North Equatorial Undercurrents (NEUCs) simulated in an eddy-resolving ocean model are evaluated, and the variability of the three NEUC jets is analyzed. This analysis provides a reference for future observations regarding how long the *in-situ* observations should be conducted to obtain a complete picture of NEUCs. We show that the primary features of the three eastward NEUC jets are fairly well reproduced by the high-resolution model of LICOM, such as the locations, tilting directions and widths of the three jets. However, the simulated NEUCs have slightly weaker magnitude and are located at shallower depths. In addition, two dominant time scales on interannual (2–7 years) and decadal (12–19 years) time scales for all three NEUC jets are found in a 39-year high-resolution LICOM simulation, although the latter is not statistically significant. The variation of these jets is related to the remarkable interannual and decadal variability in the Pacific Ocean. Our decomposition analysis indicates that both the small- and large-scale currents contribute to the total variation. Furthermore, the low-pass temporal filter of zonal velocity shows that the quasi-steady cross-basin NEUC jets can only emerge on time scales longer than the interannual periodicity.

## Introduction

With much improved observational methods, such as the Argo float, the glider and the Acoustic Doppler Current Profiler (ADCP) both on board and on subsurface moorings, increasing attention has turned to the subthermocline stationary currents in the tropical and subtropical Pacific^1–8^. In early studies based on sporadic observations, Toole *et al*.^1^ investigated the jets in the subthermocline at 10°N and 12°N, and Hu and Cui^2^ investigated zonal jets at 12°N and 18°N. These jets are named the North Equatorial Undercurrents (NEUCs) by Wang *et al*.^3^, which have been found in various observations^5–8^ recently and are typical subthermocline currents in the North Pacific Ocean.

Based on the intensified Argo floats, Qiu *et al*.^6^ systematically investigated NEUCs and presented an integrated mean structure of the NEUCs. The NEUCs have three quasi-stationary eastward zonal jets with core velocities of 2–5 cm/s, located near 8°N, 13°N and 18°N in the western Pacific Ocean basin and farther north in the eastern basin^6^. Besides, Wang *et al*.^7^ investigated the mean structure of NEUCs and the relationship between NEUCs and two other undercurrents, the Mindanao Undercurrent^9,10^ and the Luzon Undercurrent^11^, using hydrographic data. Zhang *et al*.^8^ investigated the mean structure and intraseasonal variability of the NEUCs with one-year-long ADCP measurements from four subsurface moorings deployed at 10.5°N, 13°N, 15.5°N, and 18°N, respectively, along 130°E in the Northwest Pacific.

Although a lot of fundamental characteristics have been revealed by tremendously increasing observations, our understanding of NEUCs is far from being comprehensive. We find that most studies are focused on the mean spatial structures of NEUCs using the climatological data^6,7^. However, the time mean subthermocline currents are not always stationary due to the latency of the jets^12^; for example, the amplitude of the jets is weak relative to the ambient mesoscale eddies^12^. Zhang *et al*.^8^ found that the weak eastward mean current is below the 95% confidence level in the one-year-mean ADCP data. This finding implies that one-year mean is not long enough to ensure a robust mean current. Therefore, it is worthwhile to find out how long it takes to obtain quasi-stationary NEUCs.

To answer this question, we turn to numerical simulations because it requires both high resolution and long time span to represent the spatial structure and temporal variability of NEUCs. There are only limited studies that evaluated or studied NEUCs in ocean models. Earlier studies did not focus on NEUCs, only on the striation in the subsurface^13–15^. Recently, Qiu *et al*.^16^ examined the characteristics of NEUCs in an eddy-resolving ocean model, the Ocean General Circulation Model (OGCM) for the Earth Simulator (OFES). They reported that NEUCs can be produced in the OFES but with weak magnitude. Also, using the OFES outputs, Chiang *et al*.^17^ investigated the interaction between the North Equatorial Current (NEC) and NEUCs to explain the variability of the westward propagating subthermocline eddies at the intraseasonal time scale. It is useful to investigate NEUCs simulated in different ocean models.

The purpose of the present study is to examine NEUCs and their variabilities simulated in an eddy-resolving ocean model. Our goal is to provide a reference for future observations, namely, how long the observation should be conducted to obtain quasi-steady NEUCs?

## Results

### Mean Structures of NEUCs

**Figure 1:**
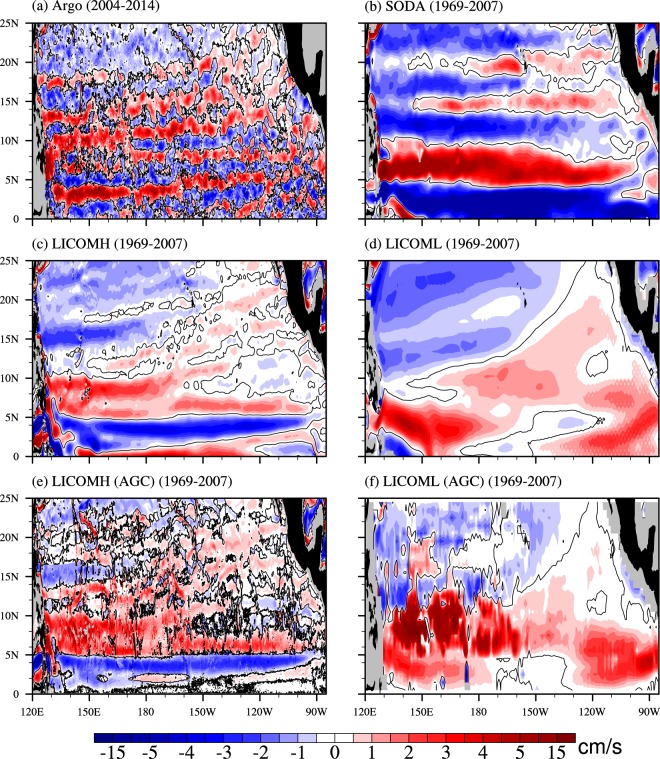
Mean zonal velocity (cm/s) on the 27.0-*σ*_*θ*_ potential density surface from (**a**) Argo (2004–2014), (**b**) SODA (1969–2007), (**c**) LICOMH (1969–2007), (**d**) LICOML (1969–2007), (**e**) LICOMH (AGC) (1969–2007) and (**f**) LICOML (AGC) (1969–2007). Zero zonal velocity is indicated by black contour. The figure was made using NCL 6.4.0 (http://www.ncl.ucar.edu/).

Figure 1 shows the climatological mean absolute geostrophic zonal velocity (AGC) on the 27.0-*σ*_*θ*_ surface based on the Argo data (Argo^18^), assimilated zonal velocity from Simple Ocean Data Assimilation Reanalysis (SODA^19^), simulated zonal velocity from the high-resolution ocean model experiment (LICOMH^20^) and the low-resolution ocean model experiment (LICOML^20^), and the AGC from LICOMH and LICOML. Three quasi-stationary jets of NEUCs defined by Qiu *et al*.^6^ can be seen clearly in Argo, located at approximately 8°N, 13°N, and 18°N near 130°–135°E, which are called the southern, middle, and northern NEUC, respectively, in the present study (Fig. 1a). We use the NEUCS, NEUCM, and NEUCN representing them, respectively, hereafter. Even though the velocity here is AGC calculated by the P-vector method (Chu^21–23^ and Yuan *et al*.^24^), the AGC shares similar structures of NEUCs displayed by the relative geostrophic velocity (see Supplementary Figs S1a and S2a–c) with the reference of 2000 m as the level of no motion following Qiu *et al*.^6^. All three jets are slightly tilted from the southwest to the northeast across the basin. The meridional width of the jet decreases from approximately 3° to 1° from the NEUCS to the NEUCN, and the clarity of the meridional structure is slightly lessened from the south to the north. The strong eastward jet at approximately 3°N comprises the northern branch of the first Tsuchiya jet or the subsurface counter current (SCC)^25^.

The zonal currents from SODA, LICOMH and LICOML, and AGC from LICOMH and LICOML are shown in Fig. 1b–f, respectively. There are also three quasi-steady eastward jets in SODA, but all three jets don’t tilt and have much greater meridional widths than those in Argo. These jets in SODA are clearly not NEUCs. Likewise, there are no NEUCs in the results of both LICOML and AGC from LICOML (Fig. 1d,f). However, the 39-year-mean LICOMH (Fig. 1c) shares similar locations, tilting directions and widths of the three jets with those in 11-year-mean Argo (Fig. 1a), but has a weaker magnitude. The weaker NEUCs in LICOMH might be caused by the longer time used for averaging, comparing 11-year mean and 39-year mean in LICOMH (see Supplementary Fig. S3), and non-geostrophic velocity in LICOMH since the 11-year-mean simulated LICOMH is still weaker than 11-year-mean Argo (Figs S3b–d vs. 1a) and AGC from LICOMH (Fig. 1e) is closer to Argo (Fig. 1a) with stronger strength than LICOMH (Fig. 1c). The main structures of NEUCs in the 39-year-mean and the 11-year-mean LICOMH are similar to each other, although there are some small differences in the strength and position due to the variability of NEUCs at decadal time scale (see Supplementary Fig. S3), which will be discussed later. From now on, the results from SODA and LICOML are no longer presented because there are no NEUCs in them. We suspect that the coarse spatial resolution of SODA and LICOML are the cause for their lack of NEUCs, since sub-grid eddies (meso-scale) are important for the formation of NEUCs^16^.

**Figure 2:**
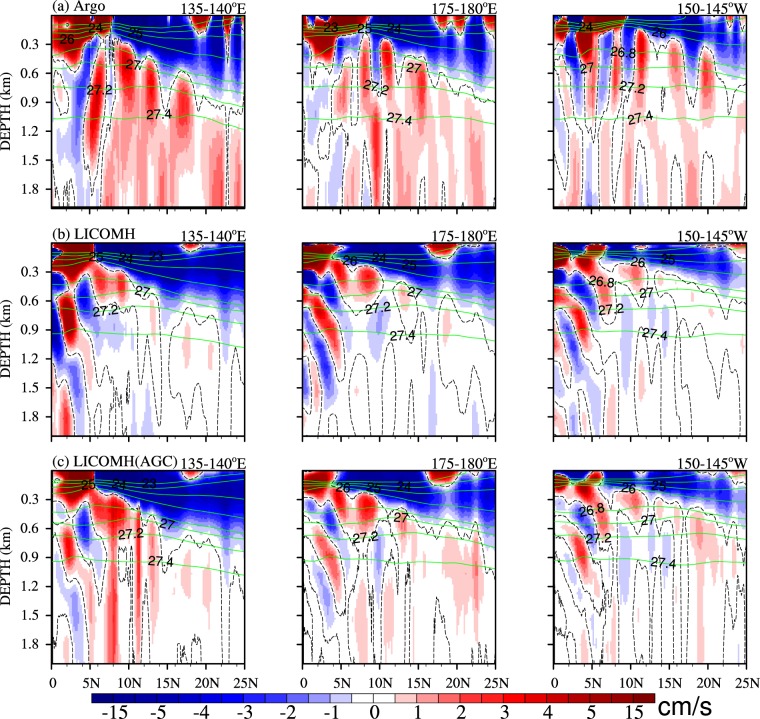
Meridional sections of mean zonal velocity (shading; cm/s) and potential density (green contour) averaged over 135°–140°E (left panels), 175°–180°E (middle), and 150°–145°W (right) for Argo (top panels), LICOMH (middle panels) and LICOMH AGC (bottom panels). Zero zonal velocity is indicated by dashed contour. The figure was made using NCL 6.4.0 (http://www.ncl.ucar.edu/).

To further investigate the characteristics of the jets in Argo, LICOMH and AGC from LICOMH, three meridional sections of NEUCs, averaged between 135°–140°E, 175°–180°E, 150°–145°W, are shown in Fig. 2. For Argo (Fig. 2a), the upper boundaries of the NEUCS, NEUCM, and NEUCN become deeper, located at 300, 450, and 600 m at the 135°–140°E section, respectively. All three NEUC jets in Argo become shallower from west to east (Fig. 2, top panels). Both LICOMH (Fig. 2, middle panels) and AGC from LICOMH (Fig. 2 bottom panels) can show those features. The mean magnitude, depth and location of NEUCs along the section averaged from 135° to 140°E in Argo, LICOMH and AGC from LICOMH (see Supplementary Table S1) are calculated as an example. The result shows that the mean magnitudes of NEUCs for AGC are closer to that for Argo, especially the southern jet, which is 1.52 cm/s for AGC, and 1.57 cm/s for Argo, while the other two jets are weaker than that for Argo. However, the depths of the three jets in Argo all reach 2000 m, while the depth of the NEUCS in LICOMH is shallower than 700 m, which may be caused by the non-geostrophic currents in LICOMH, because the NEUCS in AGC can reach 2000 m.

Based on the evaluation above, we find that LICOMH can simulate the main features of NEUCs fairly well, although the magnitude of the jets is weak, and the depths of the jets are shallow, which are partially explained by the non-geostrophic part. But the results of LICOMH and AGC in the following analysis are consistent.

### Interannual and decadal variability of NEUCs

**Figure 3:**
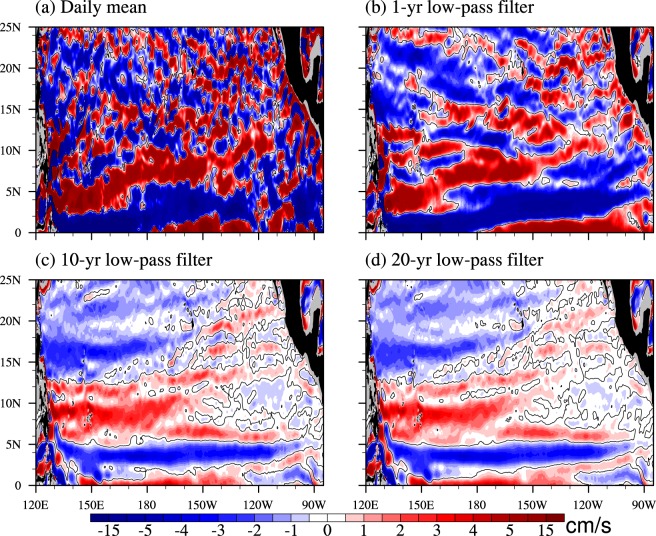
Zonal velocity (cm/s) on the 27.0-*σ*_*θ*_ potential density surface for (**a**) daily mean, (**b**) the 1-year low-pass filtered, (**c**) 10-year low-pass filtered, and (**d**) 20-year low-pass filtered results of LICOMH on December 27, 1997. The figure was made using NCL 6.4.0 (http://www.ncl.ucar.edu/).

As mentioned in the introduction, Zhang *et al*.^8^ noted that one year data is not long enough to show quasi-steady NEUC jets. The latency of NEUCs is related with strong eddies and weak currents in the ocean^12^. However, how long the observation should be conducted to obtain quasi-steady NEUCs is a practical question. To preliminarily investigate this, the daily mean and the 1-year, 10-year, and 20-year low-pass-filtered zonal currents on the 27.0-*σ*_*θ*_ surface are shown in Fig. 3. Clearly, there are no quasi-steady NEUC-like jets crossing the basin in the daily mean and 1-year low-pass-filtered fields (Fig. 3a,b). There are indeed two eastward jets at 9°N and 13°N near the western boundary in the 1-year low-pass-filtered field, but there are no NEUC-like jets east to 150°E and north to 15°N. However, the NEUC jets appear in the long-term low-pass-filtered fields (Fig. 3c,d). The NEUCS and NEUCM are clear in both 10-year and 20-year low-pass-filtered fields, also with relatively weak magnitude. These results imply that it takes nearly a decade to obtain quasi-steady NEUCS and NEUCM, but not the NEUCN, which is too weak to be identified. It is because the NEUCN on the day we chose to do analysis happens to be weak, as the NEUCN shows up on December 27, 1987 (See Supplementary Fig. S4) after 10-year and 20-year low-pass-filter. Additionally, different clarity of the NEUC jets after different filtering indicate that the variability of the NEUC jets may have different time scales, which we will discuss in the following section.

**Figure 4:**
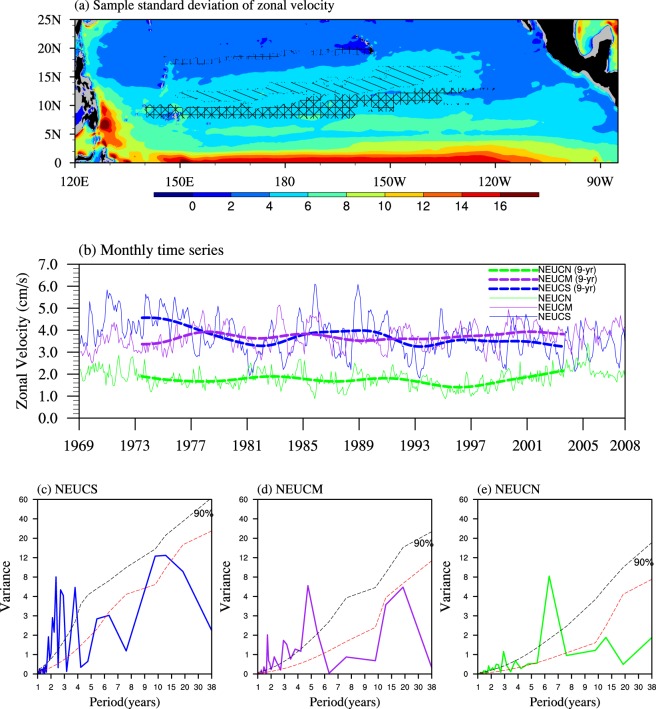
(**a**) Standard deviation of 5-day-mean zonal velocity on the 27.0-*σ*_*θ*_ potential density surface during the period 1969–2007 (shading; cm/s) and the areas for computing the three NEUCs indices (three hatched regions). (**b**) Time series of monthly indices for NEUCS (thin blue), NEUCM (thin purple) and NEUCN (thin green) in cm/s. The corresponding thick dashed curves are the 9-year low-pass filtered results. The power spectrum for (**c**) NEUCS (blue), (**d**) NEUCM (purple), and (**e**) NEUCN (green) are shown. The black and red dashed curves indicate the 90% confidence level and the red noise respectively. The figure was made using NCL 6.4.0 (http://www.ncl.ucar.edu/).

To further investigate the latency of NEUCs, we explore the variability of the NEUC jets. Here, we introduce three indices for the three jets, respectively. Each index is defined as the area mean of the eastward velocity of the jet. The areas are based on the long-term-mean eastward velocity on the 27.0-*σ*_*θ*_ surface (Fig. 1c), and are shown as hatched areas of Fig. 4a. The standard deviation (STD) of every 5-day snapshot zonal current is also computed (Fig. 4a). The magnitudes of the STDs are approximately 4–6 cm/s for the NEUCS and NEUCM and approximately 2–4 cm/s for the NEUCN. These magnitudes are larger than the magnitudes of the currents (approximately 0.5–2.0 cm/s), but are smaller than the STD in the western boundary area (approximately 15 cm/s).

The monthly mean indices are shown in Fig. 4b as the thin solid blue, purple, and green curves, representing the NEUCS, NEUCM, and NEUCN, respectively. The corresponding thick dashed curves are the 9-year low-pass-filtered values. It is clear that there are significant interannual and decadal variabilities for the NEUC jets, since the STDs of the NEUCS for the interannual and decadal variabilities are approximately 0.71 and 0.35 cm/s, respectively. The STDs of the other two indices are little bit smaller than those of the NEUCS.

To compute the period of these variabilities, spectral analysis is applied to the monthly data of the three indices after the annual cycle and the linear trend of the indices have been removed (Fig. 4c–e). The results show there are significant interannual periods of 2.5 years, 5 years, and 6.5 years for the NEUCS, NEUCM, and NEUCN, respectively. In addition, the decadal periods of 12 years, 19 years, and 13 years are found for the NEUCS, NEUCM, and NEUCN, respectively, although all peaks are below the 90% confidence level. Therefore, to obtain quasi-steady NEUC jets while avoiding the latency, we believe that the dominant variabilities above the 90% confidence level on the interannual time scale should be included in the averaging time span. That is, the period of observations for NEUCs need to last for years or even close to a decade. However, further testing is needed to quantify the exact time length, which is discussed in the Summary and Discussion.

Since the El Niño-Southern Oscillation (ENSO) and Pacific Decadal Oscillation (PDO) are the typical interannual and decadal variability in the Pacific Ocean, respectively, the lag-lead cross correlations are employed to the annual three NEUCs indices and the Niño 3.4 index, as well as the PDO index (see Supplementary Fig. S5c,d). For the relationship between NEUCs and Niño 3.4 (see Supplementary Fig. S5a,d), the NEUCs indices have minimum negative correlation coefficients of −0.31, −0.16, and −0.39 at zero lag for the NEUCS, NEUCM, and NEUCN, respectively. This means the NEUC jets become weaker during the El Niño and stronger during the La Niña, but the correlation coefficients are relatively small, especially for the NEUCM, which is below the 95% significance level. For the decadal variability (see Supplementary Fig. S5b,c), we find that the NEUCS and NEUCN display minimum negative correlation coefficients of −0.61 and −0.34 at zero lag, while the NEUCM shows a positive maximum coefficient of 0.40 with one-year lag behind the PDO index. This means the NEUCS and NEUCN become weaker (stronger) during the positive (negative) phase of the PDO. The opposite characteristics between the NEUCS (or NEUCN) and NEUCM is related to the change of background currents, which will be discussed later.

**Figure 5:**
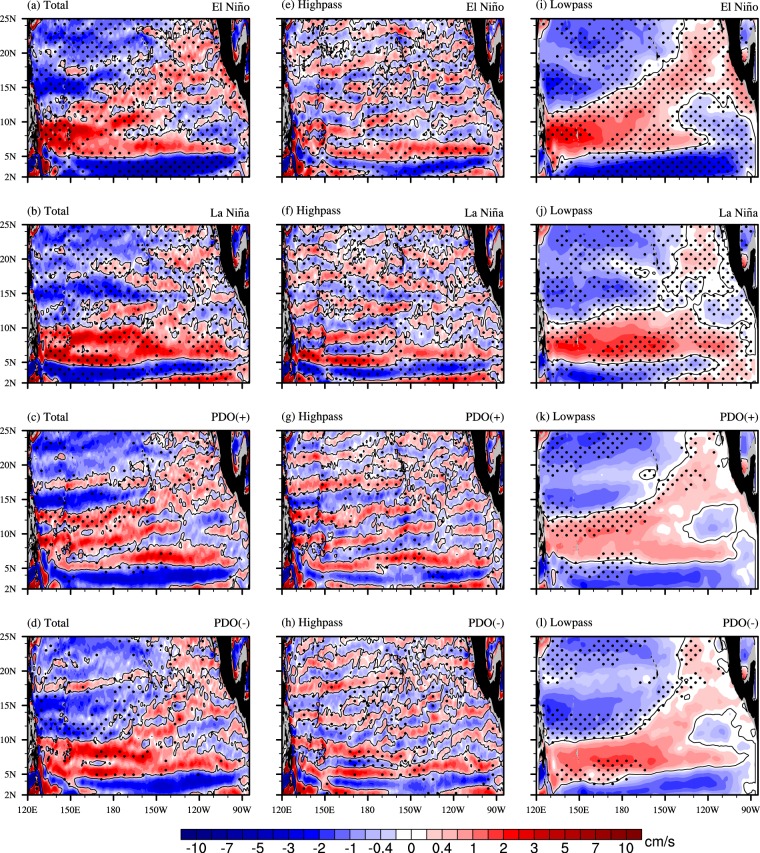
Composition of mean zonal velocity on the 27.0-*σ*_*θ*_ potential density surface for (**a**) positive ENSO phase (El Niño) and (**b**) negative ENSO phase (La Niña). The positive (negative) events are defined by the ONI^28^. (**e** and **i**) are decompositions of (a) by using high- and low-pass spatial filters on 4° × 4° grid, respectively. (**f** and **j**) are the same as (**e** and **i**), except for (**b**). Stippling indicates the 95% confidence level. The two lower panels are the same as the two upper ones, except for PDO. The positive and negative PDO phases are (1981–1988) and (1969–1976), respectively. The figure was made using NCL 6.4.0 (http://www.ncl.ucar.edu/).

To further investigate the interannual and decadal variability of NEUCs, the compositions of positive and negative phases of ENSO and PDO are examined. Figure 5a,b show the mean zonal velocity on the 27.0-*σ*_*θ*_ surface during El Niño and La Niña events, respectively. There are no three NEUC zonal jets in the basin for both phases of ENSO, except for the NEUCS. This result is consistent with the low correlations between the NEUCs indices and Niño 3.4 index. It also suggests that the NEUCs indices and the Niño 3.4 index are negatively correlated, since the existing NEUC jets are strong (weak) during La Niña (El Niño).

The compositions are further decomposed into small- and large-scale circulations using the high-pass and low-pass spatial filters, respectively. We find that the variability on the interannual time scale is affected by both small- and large-scale currents. In the high-pass-filtered currents (Fig. 5e,f), there are clearly multiple alternating zonal jets, also negatively correlated with the Niño 3.4 index. However, in the low-pass-filtered currents (Fig. 5i,j), there is an eastward flow in the south and a westward flow in the north over the NEUCs latitudes (5°–20°N), with the boundary at approximately 10°–15°N tilting from southwest to northeast. The spatial pattern of low-pass-filtered currents is similar to the simulated results in LICOML (Fig. 1d), which implies that the low-pass-filtered circulation is dominated by the large-scale wind-driven circulation. The NEUCM and NEUCN are in the region of westward currents, while the NEUCS is in the region of eastward currents. Therefore, the NEUCM and NEUCN can be canceled out by the large-scale currents, while the NEUCS can be enhanced. That explains why the two northern jets are weak. It is also clear that the large-scale currents have significant interannual variation (above the 95% confidence level), as the center of the eastward currents has weaker strength during the La Niña phase than during the El Niño phase. That outcome will further enhance the existing NEUC jets in the central Pacific during La Niña.

For the PDO, Fig. 5c,d show the mean zonal velocity on the 27.0-*σ*_*θ*_ surface during the positive and negative phases of the PDO, respectively. The jets are much clearer on the decadal time scale than on the interannual time scale (Fig. 5a,b). This is consistent with the relatively higher correlations between the NEUCs indices and PDO index. However, NEUCs in the decadal time scale are much more complicated. As we just mentioned, that NEUCM is positively correlated with the PDO index, while the NEUCS and NEUCN are negatively correlated with the PDO index. Those can also be clearly seen in the composite pattern. The NEUCM is strong during the positive phase, but disappears during the negative phase. For the NECUS and NEUCN, they are weak (strong) during the positive (negative) phase.

To understand the mechanism of the jets on the decadal time scale, we also decompose currents into small- and large-scale circulations using the high-pass and low-pass spatial filters, respectively. Both the large- and small-scale currents contribute to the total variability. The small-scale circulation has significant variability above the 95% confidence level in NEUCs areas: weak during positive phase and strong during negative phase for the southern and northern branches, and opposite correlation for the middle branch (Fig. 5g,h).

The spatial pattern of large-scale currents on the decadal time scale (Fig. 5k,l) is generally similar to that on the interannual time scale (Fig. 5i,j) and to that in the low-resolution results (Fig. 1d). Both magnitude and location of large-scale currents on the decadal time scale have significant changes (Fig. 5k,l). The centers of the eastward and westward currents are located at 10°N and 15°N with a magnitude of approximately 1 cm/s in the positive phase, while they both become slightly stronger (approximately about 0.4 cm/s) and shift southward (by 2–3°) in the negative phase. This variation is large enough to damp the NEUCM and enhance the NEUCS. That explains why the variations of the NEUCS and NEUCM are out of phase. It is interesting that the NEUCN only occurs in a region with weak westward currents. As a result, the NEUCN does not have any effect from the large-scale currents and has the same phase as the NEUCS.

In summary, we find two primary time scales in the NEUC jets: interannual (approximately 2- to 7-year) and decadal (approximately 13- to 19-year), although the latter is below the 90% confidence level. The periods on the interannual time scale become longer with the latitude: 2.5 years, 5 years, and 6.5 years for the NEUCS, NEUCM, and NEUCN, respectively. In addition, we find that the correlation coefficient between PDO index and the NEUCM is different from those of the other two jets with the PDO index. The decomposition analysis indicates that both small- and large-scale currents contribute to the total variation, and the westward large-scale currents north to 12°N are responsible for the phase difference between NEUCS and NEUCM. The variability of the large-scale currents will offset the change of the small-scale jets for the NEUCM.

## Summary and Discussion

In the present paper, the mean states of NEUC jets simulated in an eddy-resolving ocean model are evaluated first. Using the Argo data as truth, we conclude that the primary features of NEUCs can be well reproduced by LICOMH, such as main locations, tilting directions, and widths of the three jets. However, the simulated jets of the NEUCs have slightly weak magnitude and shallower depths. We also believe that the horizontal resolution of the numerical model is the essential factor for the NEUCs simulation by comparing with two coarse resolution datasets.

Then, variabilities of the three NEUC jets are investigated. We find two time scales in the NEUC jets: interannual (approximately 2 to 7 years) and decadal (approximately 13 to 19 years), although the latter is below the 90% confidence level. Those variations of the jets are related to the remarkable interannual and decadal variability in the Pacific Ocean. In addition, we find that the periods on the interannual time scale become longer with the latitude: 2.5 years, 5 years, and 6.5 year for the NEUCS, NEUCM, and NEUCN, respectively. According to the generation mechanism of NEUCs proposed by Qiu *et al*.^16^ in which the NEUC jets are formed through the baroclinic Rossby wave triad interaction theory, it is not surprising that the northern jets of the NEUCs have longer periods, because the period of Rossby wave is inversely proportional to the Coriolis parameter (*f*).

The composite and correlation analyses indicate that the NEUCs indices are negatively correlated with the ENSO phases. Also, the southern and northern jets are negatively correlated with the PDO phases, and with larger correlation coefficients, while the correlation coefficient between NEUCM and PDO is positive. The decomposition analysis indicates that both the small- and large-scale currents contribute to the total variation. The opposite phases between the correlation of NEUCM with PDO and the correlations of the other two jets with PDO is mainly due to the westward large-scale currents dominate the total variation and offset the changes of the small-scale jets. However, the mechanisms of ENSO and PDO affecting NEUCs need further investigation. The surface wind stress may be an important candidate to connect NEUCs and dominant climate variability, since the wind stress shows significant correlation to NEUCs at the decadal time scale (see Supplementary Figs S6–8), just as the relationship between NEUCs and PDO.

Although a theory of the NEUCs dynamics has been proposed by Qiu *et al*.^16^, there is no theory about the variations of NEUCs. The decomposition analysis of the variability (Fig. 5) can support the theory of Qiu *et al*.^16^ on one hand, and can clarify the role of large-scale currents in the variation of NEUCs on the other hand. The small-scale alternating zonal jets (Fig. 5e–h) have been explored in a comprehensive way in previous studies. These studies found that the alternating zonal jets in the ocean are caused by the propagation of both anticyclonic and cyclonic eddies, which are triggered by the instability radiated from the eastern boundary (Kamenkovich *et al*.^26^; Wang *et al*.^27^; Reilly *et al*.^28^; Buckingham and Cornillon^29^). The mechanism of the alternating zonal jets is similar to that of NEUCs, which also indicates that the spatial high-pass-filtered part can be explained by the theory of Qiu *et al*.^16^. On the other hand, the large-scale circulation also plays an important role in the variation of NEUCs. The westward large-scale circulation may cancel out the eastward zonal jets to the north of the zero contour and enhance the eastward zonal jets to the south (Fig. 5k,l). That is also the reason why the NEUCM is in the opposite phase of the NEUCS and NEUCN at the decadal time scale. The large-scale circulation plays a role as the background circulation to modify the alternating zonal jets and their variation.

**Figure 6:**
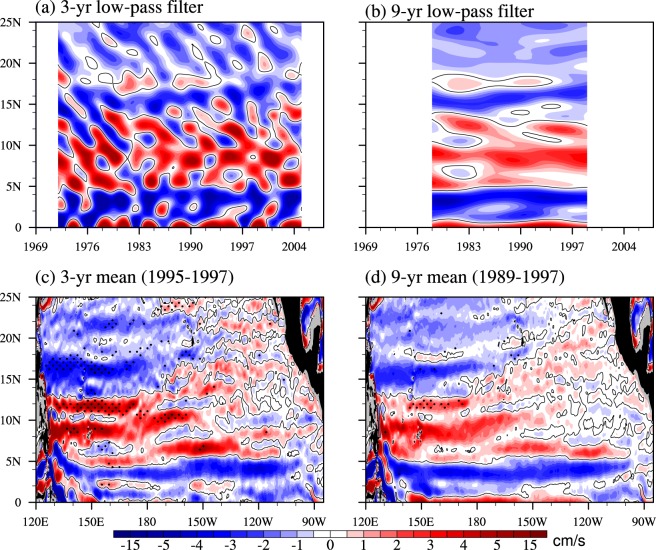
Hovmoller diagrams for (**a**) 3-year and (**b**) 9-year low-pass-filtered zonal velocity on the 27.0-*σ*_*θ*_ potential density surface averaged over 150°E–180°. (**c**) 3-year and (**d**) 9-year mean zonal velocity on the 27.0-*σ*_*θ*_ potential density surface averaged over 1995–1997 and 1989–1997 respectively. The stippling indicates the 90% confidence level. Only the zero contour is shown. Units: cm/s. The figure was made using NCL 6.4.0 (http://www.ncl.ucar.edu/).

To answer the question of how long it will take to obtain quasi-steady NEUC jets, we further investigate the sensitivity of zonal currents to different time scales and find that nearly a decade (longer than the significant periods of NEUCs) is necessary to obtain quasi-steady NEUC jets in the eddy active ocean. Based on the major periodicity of interannual variability, the 3-year and 9-year low-pass-filter are applied to the time series of zonal velocity to examine the appearance of NEUCs. Figure 6a,b are the Hovmoller diagrams for 3-year and 9-year low-pass-filtered zonal currents on the 27.0-*σ*_*θ*_ surface. Comparing with the 3-year low-pass filtered values, the 9-year filtered values are relatively consistent at the latitudes of three NEUC jets, which implies the 9-year mean can represent a steady pattern of NEUCs. Furthermore, to inspect the horizontal pattern of NEUCs, the randomly chosen 3-year (1995–1997) and 9-year (1989–1997) original time series are averaged and the Student’s t-test are applied to show the significant differences verses the climatological mean (Fig. 6c,d). The 3-year mean values of NEUCM and NEUCS are significant different (90% confidence level) from the climatological values, while the difference between the 9-year mean values and climatological means are less significant than the 3-year values. Moreover, the spatial correlation coefficient between the 9-year mean and the climatological values over the western and central Pacific region (130°E~130°W, 5°N~20°N) is 0.89, while it is only 0.73 for the 3-year mean, which indicates the 9-year mean is much steadier. So, the answer of the question at the beginning is that the quasi-steady cross-basin NEUC jets can only emerge on time scales longer than the interannual periodicity, near one decade.

## Methods

### The absolute geostrophic currents (AGC)

The P-vector method and the thermal wind relation are applied to obtain the AGC velocity from the Argo data following Chu^21–23^ and Yuan *et al*.^24^. The thermal wind vector formulas are as follows:

1 $$\frac{\partial u}{\partial z}=\frac{g}{\rho f}\frac{\partial \rho }{\partial y}$$

2 $$\frac{\partial v}{\partial z}=-\,\frac{g}{\rho f}\frac{\partial \rho }{\partial x}$$

where u and v are the zonal and meridional velocities, respectively; x, y, and z are the zonal, meridional, and vertical coordinates, respectively; ρ is seawater density; g is gravity; and f is the Coriolis parameter.

The P-vector method is based on the conservations of potential density and potential vorticity and on two approximations of the geostrophic balance and the Boussinesq approximation. As a result, a unit vector P can be defined as:

3 $${\rm{P}}=\frac{\nabla {\rm{\rho }}\times \nabla {\rm{q}}}{|\nabla {\rm{\rho }}\times \nabla {\rm{q}}|},$$

which is the intersection of isopycnal and equal potential vorticity surfaces, where ρ is seawater density and q is potential vorticity. The AGC is assumed to follow the unit vector P, that is,

4 $${\rm{V}}=r(x,y,z)P$$

where *r* is the proportionality coefficient. The thermal wind relation at two depths can determine *r*.

### Temporal and spatial filters

Running mean is employed in the present study for the temporal and spatial filtering. The temporal filter is based on the Lanczos filter weight^30^, which is a commonly used filtering technique to highlight variability at different scales. The spatial filter uses a bin of 4° × 4°, as in Maximenko *et al*.^13,31^ and Lu *et al*.^32^, to separate the large- and small-scale currents by running average over the bin.

### Niño 3.4 index

The commonly used Niño 3.4 index associated with ENSO is defined by the area average of monthly SST anomalies in the region of (5°S–5°N, 170°W–120°W)^33^. The climatology is derived from the LICOMH experiment with the same period of the NEUC jets from 1969 to 2007. The El Niño (La Niña) is characterized by five consecutive 3-month running mean SST anomalies in the Niño 3.4 region that is above (below) the threshold of +0.5 °C (−0.5 °C). This standard of measure is known as the Oceanic Niño Index (ONI).

### PDO index

The monthly PDO index used here is defined by the time series of the leading empirical orthogonal function (EOF) of monthly mean sea surface temperature (SST) anomalies for the Pacific Ocean north of 20°N^34^. The SST field is from the eddy-resolving ocean model LICOMH experiment from 1948 to 2007. The PDO index used here is from 1969 to 2007 to correspond to the period of the NEUC jets.

### Numerical experiments

Two ocean model hindcast experiments with different spatial resolutions are used in the present study. The ocean model used here was developed at the Sate Key Laboratory of Numerical Modeling for Atmospheric Sciences and Geophysical Fluid Dynamics (LASG), the Institute of Atmospheric Physics (IAP). The model is named the LASG/IAP Climate system Ocean Model version 2.0 (LICOM2.0)^20^. The LICOMH experiment is an eddy-resolving version with a 0.1° × 0.1° horizontal grid and 55 vertical levels. In the upper 300 m, 36 uneven levels are used, and every layer thickness is less than 10 m. The model domain covers only 79°S–66°N, excluding the Arctic Ocean to avoid the singularity of the North Pole in the longitude-latitude grid. After the 12-year spin-up, a 60-year hindcast experiment was conducted using the daily Coordinated Ocean-Ice Reference Experiments (COREs) data from 1948 to 2007 (Large and Yeager^35^) as the forcing. In addition, biharmonic viscosity and diffusivity schemes are used in the momentum and tracer equations, respectively. The LICOML experiment has 1° × 1° horizontal resolution and 30 vertical levels. The experiment was also run using the daily COREs data, but with a much longer spin-up time (500 years). The parameterization of eddies from Gent and McWilliams (1990) is applied in LICOML, which is turned off in LICOMH. The temperature and salinity during 1969–2007 from the two experiments are used in the present study. The ENSO and PDO are well simulated by LICOMH (see Supplementary Fig. S9).

### Observation and assimilation datasets

Two datasets are used in the present study to validate the model outputs: (1) the Roemmich-Gilson Argo Climatology data^18^ (http://sio-argo.ucsd.edu/RG_Climatology.html), in which the Argo data from 2004 to 2014 have been interpolated onto the regular 1/6° × 1/6° grid with 58 vertical levels by using a weighted least-squares fit; (2) Simple Ocean Data Assimilation Reanalysis (SODA) Version 2.0.2-4 with the horizontal resolution of 0.5° × 0.5° from 1969 to 2007^19^ (http://iridl.ldeo.columbia.edu/SOURCES/.CARTON-GIESE/.SODA/.v2p0p2-4/).

## Electronic supplementary material

Supplementary Information

## References

[1] Toole JM, Zou E, Millard R (1988). On the circulation of the upper waters in the western equatorial Pacific Ocean. Deep. Sea. Res. I..

[2] Hu D, Cui M, Qu T, Li Y (1991). A subsurface northward current off Mindanao identified by dynamic calculation. Elsevier Oceanogr. Ser..

[3] Wang F, Hu D, Bai H (1998). Western boundary undercurrents east of the Philippines. Proceedings of the 4th Pacific Ocean Remote Sensing Conference (PORSEC)..

[4] Qiu B, Joyce T (1992). Interannual variability in the mid-and low-latitude western North Pacific. J. Phys. Oceanogr..

[5] Cravatte S, Kessler W, Marin F (2012). Intermediate zonal jets in the tropical Pacific Ocean observed by Argo floats. J. Phys. Oceanogr..

[6] Qiu B, Rudnick D, Chen S, Kashino Y (2013). Quasi-stationary North Equatorial Undercurrent jets across the tropical North Pacific Ocean. Geophys. Res. Lett..

[7] Wang F, Zang N, Li Y, Hu D (2015). On the subsurface countercurrents in the Philippine Sea. J. Geophys. Res. Oceans..

[8] Zhang, L. *et al*. Structure and variability of the North EquatorialCurrent/Undercurrent from mooring measurements at 130°E in the Western Pacific. *Scientific Reports*. **7**, 10.1038/srep46310 (2017). 10.1038/srep46310 PMC5395815 28422095

[9] Wang F, Hu D (1998). Dynamic and thermohaline properties of the Mindanao Undercurrent, part I: Dynamic structure. Chin. J. Oceanol. Limnol..

[10] Wang F, Hu D (1998). Dynamic and thermohaline properties of the Mindanao Undercurrent, part II: Thermohaline structure. Chin. J. Oceanol. Limnol..

[11] Hu D (2013). Direct Measurements of the Luzon Undercurrent. J. Phys. Oceanogr..

[12] Berloff P, Karabasov S, Farrar J, Kamenkovich I (2011). On latency of multiple zonal jets in the oceans. J. Fluid. Mech..

[13] Maximenko N, Bang B, Sasaki H (2005). Observational evidence of alternating zonal jets in the world ocean. Geophys. Res. Lett..

[14] Nakano H, Hasumi H (2005). A series of zonal jets embedded in the broad zonal flows in the Pacific obtained in eddy-permitting ocean general circulation models. J. Phys. Oceanogr..

[15] Richards K, Maximenko N, Bryan F, Sasaki H (2006). Zonal jets in the Pacific Ocean. Geophys. Res. Lett..

[16] Qiu B, Chen S, Sasaki H (2013). Generation of the North Equatorial Undercurrent jets by triad baroclinic Rossby wave interactions. J. Phys. Oceanogr..

[17] Chiang T, Wu C, Qu T, Hsin Y (2015). Activities of 50–80 day subthermocline eddies near the Philippine coast. J. Geophys. Res. Oceans..

[18] Roemmich D, Gilson J (2009). The 2004–2008 mean and annual cycle of temperature, salinity, and steric height in the global ocean from the Argo Program. Prog. Oceanogr..

[19] Carton JA, Giese BS (2008). A reanalysis of ocean climate using Simple Ocean Data Assimilation (SODA). Mon. Weather. Rev..

[20] Liu H, Lin P, Yu Y, Zhang X (2012). The baseline evaluation of LASG/IAP climate system ocean model (LICOM) version 2. Acta. Meteorol. Sin..

[21] Chu P (1995). P-vector method for determining absolute velocity from hydrographic data. Mar. Technol. Soc. J..

[22] Chu P (2000). P-vector spiral and determination of absolute velocities. J. Oceanogr..

[23] Chu Peter C. (2006). P-Vector Inverse Method.

[24] Yuan D, Zhang Z, Chu P, Dewar W (2014). Geostrophic circulation in the tropical north Pacific Ocean based on Argo profiles. J. Phys. Oceanogr..

[25] Cravatte S, Kestenare E, Marin F, Dutrieux P, Firing E (2017). Subthermocline and intermediate zonal currents in the tropical Pacific Ocean: paths and vertical structure. J. Phys. Oceanogr..

[26] Kamenkovich I, Berloff P, Pedlosky J (2009). Role of Eddy Forcing in the Dynamics of Multiple Zonal Jets in a Model of the North Atlantic. J. Phys. Oceanogr..

[27] Wang J, Spall M, Flierl G, Malanotte-Rizzoli P (2012). A new mechanism for the generation of quasi-zonal jets in the ocean. Geophys. Res. Lett..

[28] O’Reilly C, Czaja A, LaCasce J (2012). The emergence of zonal ocean jets under large-scale stochastic wind forcing. Geophys. Res. Lett..

[29] Buckingham C, Cornillon P (2013). The contribution of eddies to striations in absolute dynamic topography. J. Geophys. Res. Oceans..

[30] Duchon CE (1979). Lanczos filtering in one and two dimensions. J. Appl. Meteor..

[31] Maximenko N, Melnichenko O, Niiler P, Sasaki H (2008). Stationary mesoscale jet-like features in the ocean. Geophys. Res. Lett..

[32] Lu J, Wang F, Liu H, Lin P (2016). Stationary mesoscale eddies, upgradient eddy fluxes, and the anisotropy of eddy diffusivity. Geophys. Res. Lett..

[33] Trenberth, K. E., Caron, J. M., Stepaniak, D. P. & Worley, S. Evolution of El Niño–Southern Oscillation and global atmospheric surface temperatures. *J*. *Geophys*. *Res*. *Atmos*. **107****(**D8**)**, 10.1029/2000JD000298 (2002).

[34] Mantua NJ, Hare SR, Zhang Y, Wallace JM, Francis RC (1997). A Pacific interdecadal climate oscillation with impacts on salmon production. Bull. Amer. Meteor. Soc..

[35] Large, W. & Yeager, S. Diurnal to decadal global forcing for ocean and sea-ice models: The data sets and flux climatologies. *NCAR Technical Note NCAR*/*TN-460*+*STR*, pp. 111, 10.5065/D6KK98Q6 (2004).

