# Supplementary material for: Interannual and decadal variability of the North Equatorial Undercurrents in an eddy-resolving ocean model
Source: Sci Rep. 2018 Nov 20;8:17112. doi: 10.1038/s41598-018-35469-2 (PMC6244160; doi:10.1038/s41598-018-35469-2)
Supplement: Supplementary file 1 — Supplementary Information [file 41598_2018_35469_MOESM1_ESM.pdf]

Supplementary Information:

Interannual and decadal variability of the North Equatorial Undercurrents in an eddy-resolving ocean model

Yiwen Li[1,2], Hailong Liu[1, 2*], Pengfei Lin[1, 2]

1 State Key Laboratory of Numerical Modeling for Atmospheric Sciences and Geophysical Fluid Dynamics, Institute of Atmospheric Physics, Chinese Academy of Sciences, Beijing 100029, China

2 College of Earth Sciences, University of Chinese Academy of Sciences, Beijing 100049, China

* Corresponding author: Hailong Liu (lhl@lasg.iap.ac.cn)

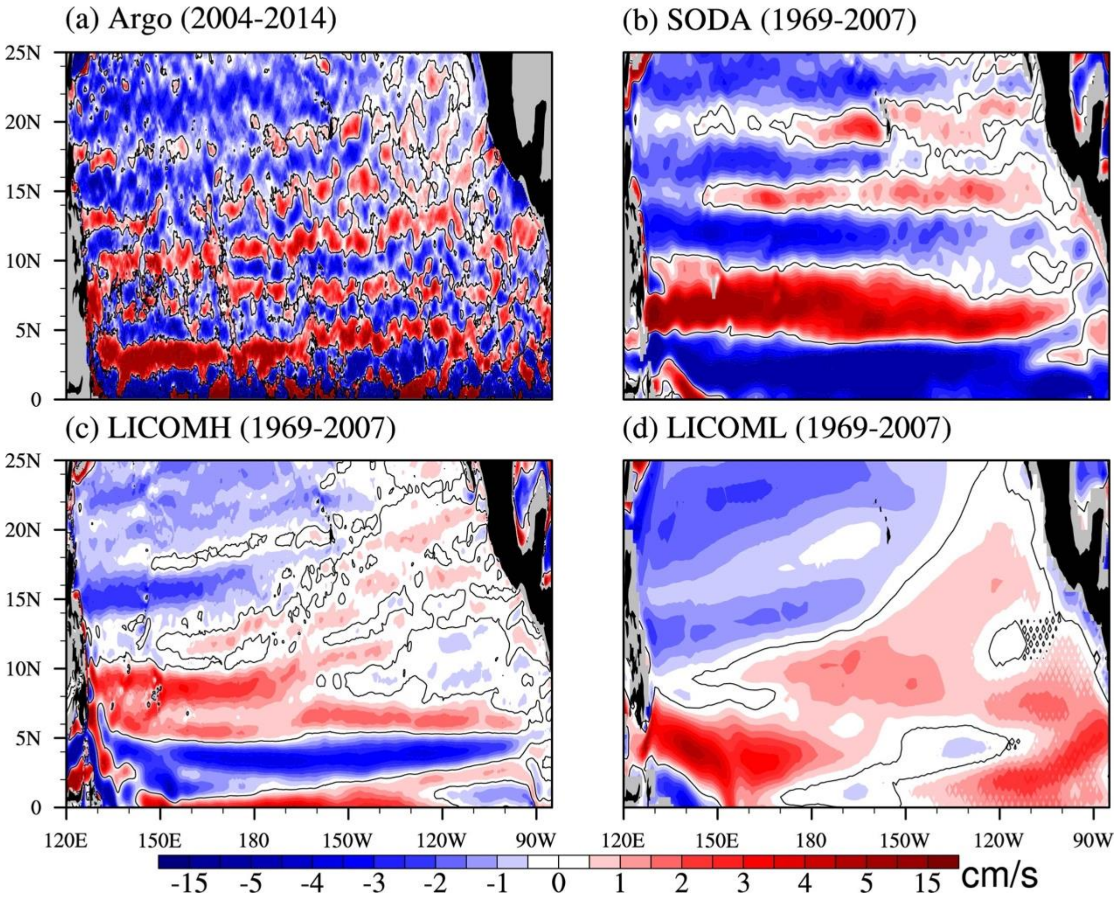

Figure S1. Mean zonal velocity (cm/s) on the 27.0-σ_θ potential density surface from (a) Argo (2004-2014)27, (b) SODA (1969-2007)28, (c) LICOMH (1969-2007), and (d) LICOML (1969-2007). Zero zonal velocity is indicated by black contour. The figure was made using NCL 6.4.0 (http://www.ncl.ucar.edu/).

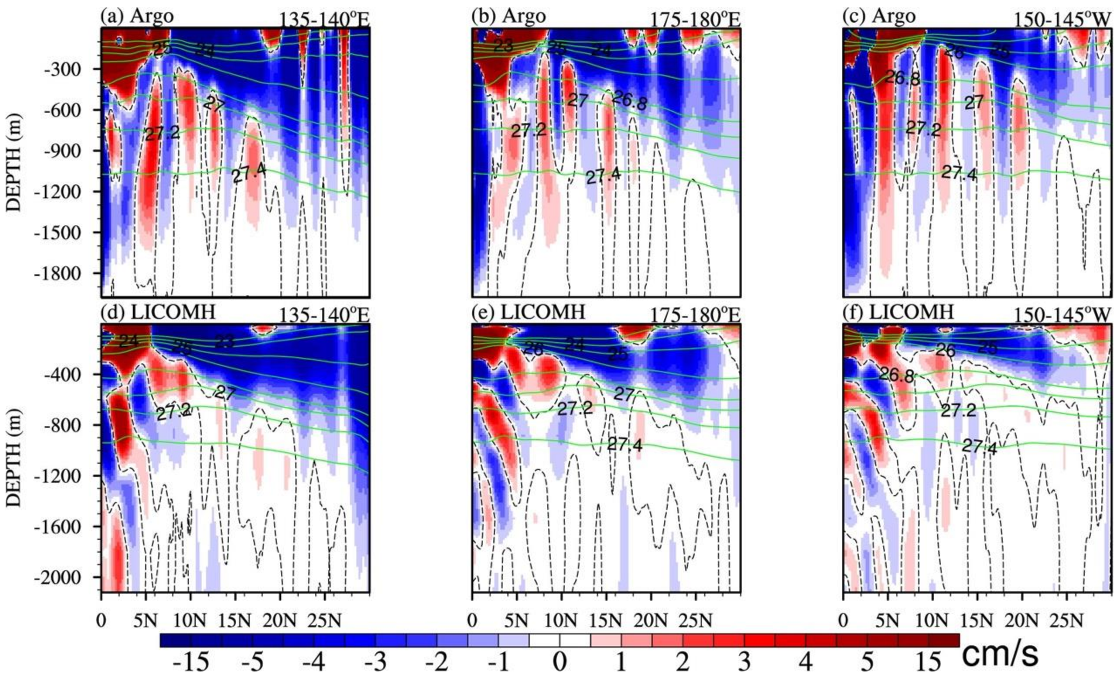

Figure S2. Meridional sections of mean zonal velocity (shading; cm/s) and potential density (green contour) averaged over 135°-140°E (left panels), 175°-180°E (middle), and 150°-145°W (right) for Argo27 (top panels) and LICOMH (bottom panels). Zero zonal velocity is indicated by dashed contour. The figure was made using NCL 6.4.0 (http://www.ncl.ucar.edu/).

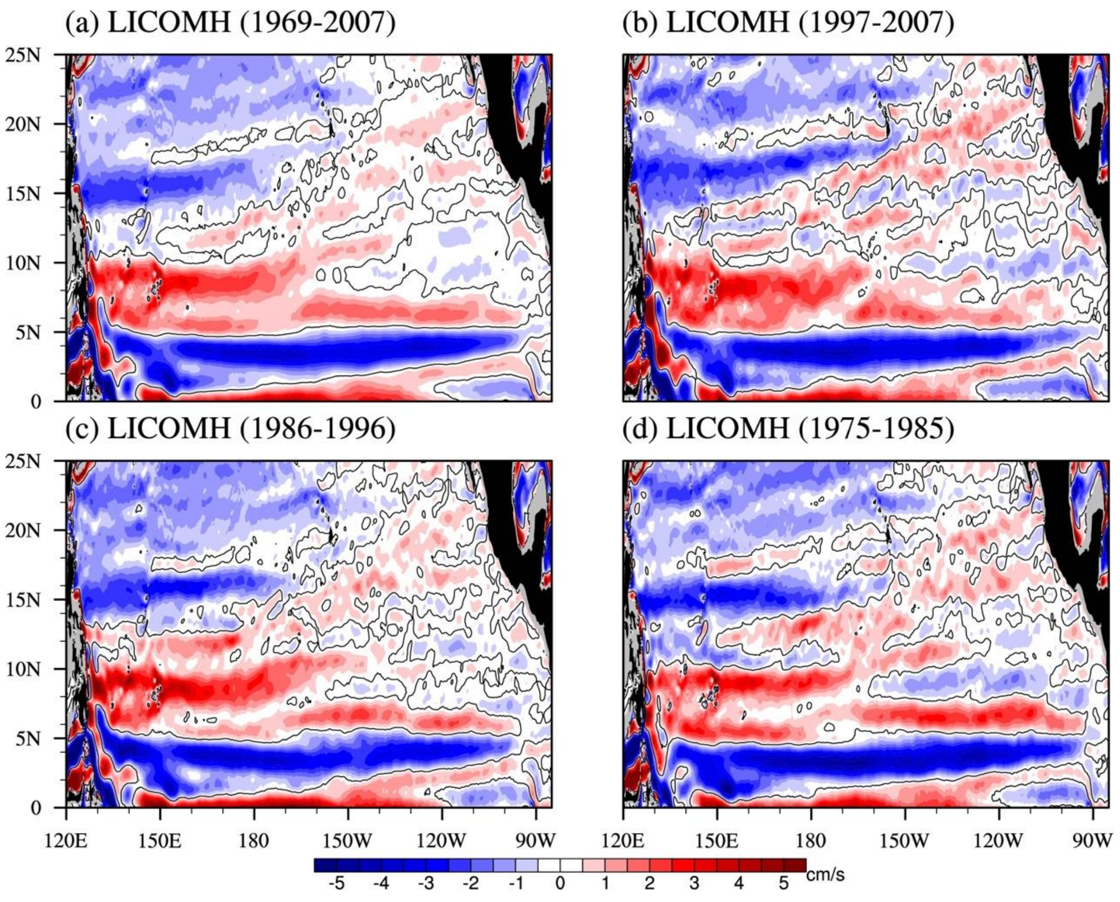

Figure S3. Mean zonal velocity (cm/s) on the 27.0-σ_θpotential density surface from LICOMH during (a) 1969-2007, (b) 1997-2007, (c) 1986-1996, and (d) 1975-1985. Zero zonal velocity is indicated by black contour. The figure was made using NCL 6.4.0 (http://www.ncl.ucar.edu/).

		**NEUCS**	**NEUCM**	**NEUCN**
**Argo**	Magnitude (cm/s)	1.57	1.29	1.29
	Depth (m)	300~2000	400~2000	600~2000
	Location (°N)	8.5~11	12~14	16~18
**LICOMH**	Magnitude (cm/s)	1.15	0.29	0.30
	Depth (m)	300~700	500~1800	700~1800
	Location (°N)	8~10	12~14	16.5~19
**LICOMH AGC**	Magnitude (cm/s)	1.52	0.67	0.32
	Depth (m)	300~2000	500~2000	700~2000
	Location (°N)	8~10	12~14	16.5~19

Table S1. The mean magnitude, depth and location of three jets of NEUCs averaged 135°-140°E section for Argo, LICOMH and LICOMH AGC. The mean strength are averaged eastward zonal velocity of three NEUCs from 135°-140°E section below 200m between the locations of three NEUCs, respectively. Depth is the top and bottom edge of eastward NEUCs.

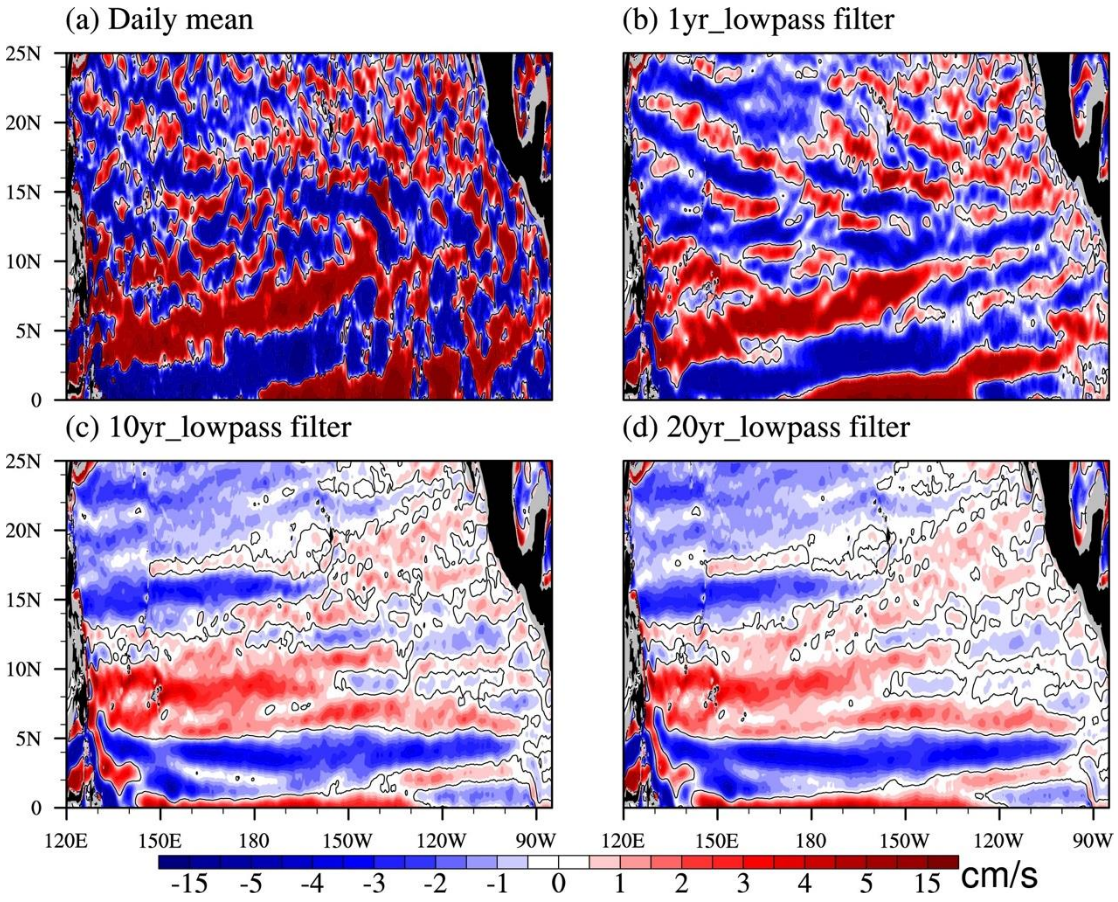

Figure S4. Zonal velocity (cm/s) on the 27.0-σ_θ potential density surface for (a) daily mean, (b) the 1-year low-pass filtered, (c) 10-year low-pass filtered, and (d) 20-year low-pass filtered results of LICOMH on December 27, 1987. The figure was made using NCL 6.4.0 (http://www.ncl.ucar.edu/).

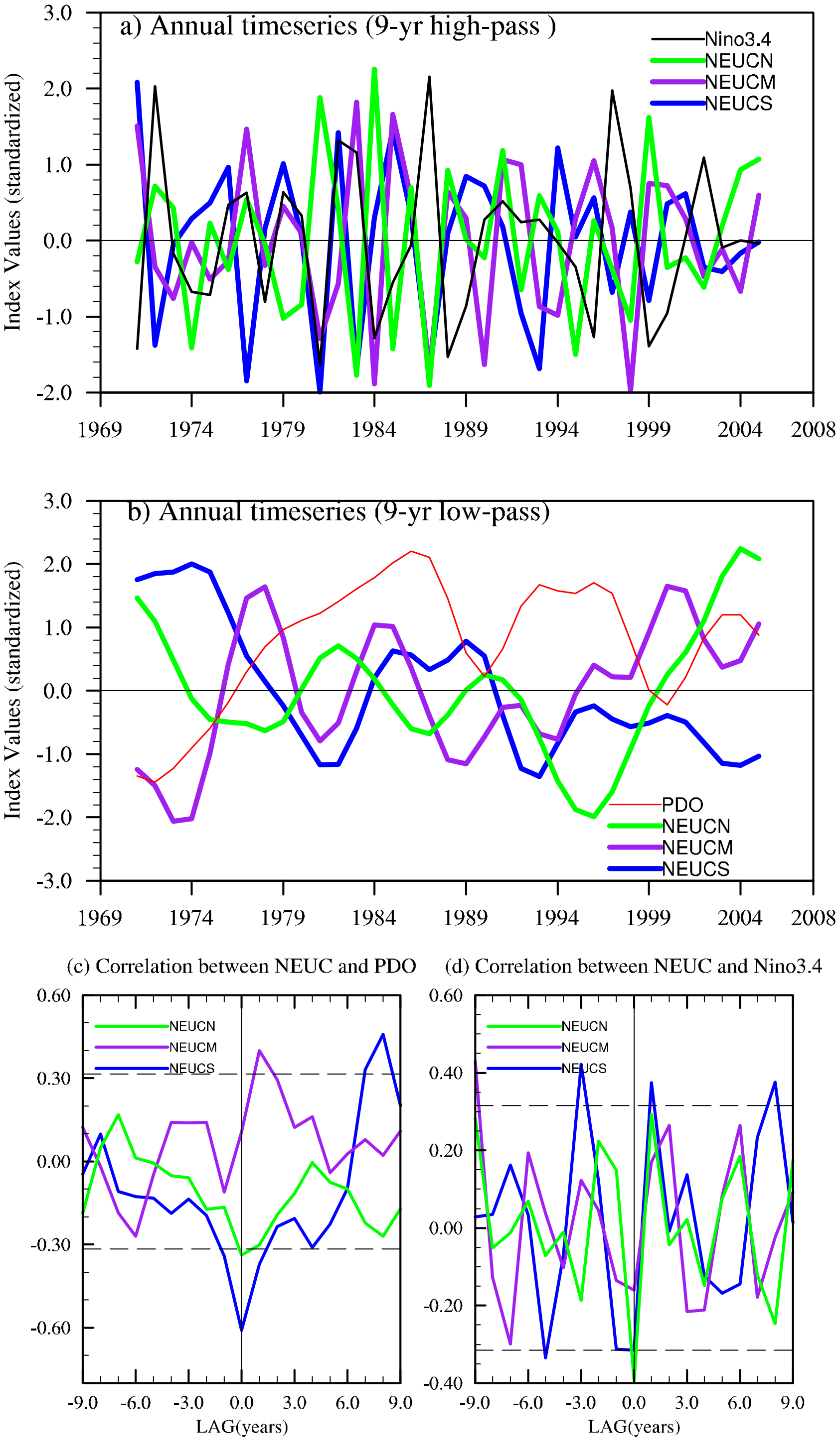

Figure S5. (a) The 9-year high-pass-filtered annual normalized indices for the NEUCS (blue), NEUCM (purple), and NEUCN (green) in cm/s. The black curve is the 9-year high-pass-filtered annual normalized Nino3.4 index. (b) shows the 9-year low-pass-filtered annual normalized indices. The red curve is the PDO index. (c) shows the lag-lead cross correlations between three NEUCs indices and PDO index. (d) is the same as (c), except for the Nino3.4 index. The dashed curves indicate the 95% significance level. The figure was made using NCL 6.4.0 (http://www.ncl.ucar.edu/).

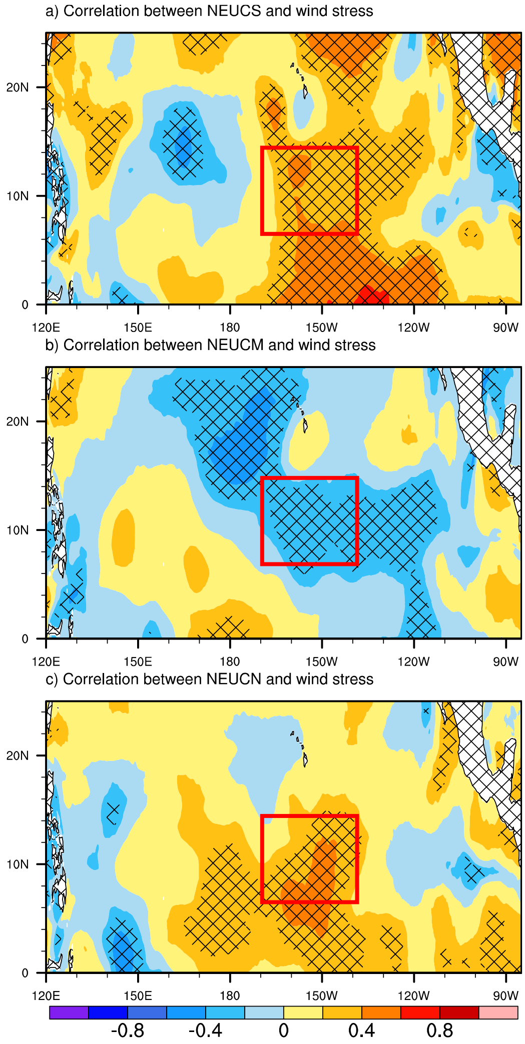

Figure S6. The correlation coefficients of surface wind stress with a) the NEUCS, b) NEUCM, and c) NEUCN. Hatched areas indicate the 95% confidence level. The red boxes are the regions where the wind stresses are averaged. The figure was made using NCL 6.4.0 (http://www.ncl.ucar.edu/).

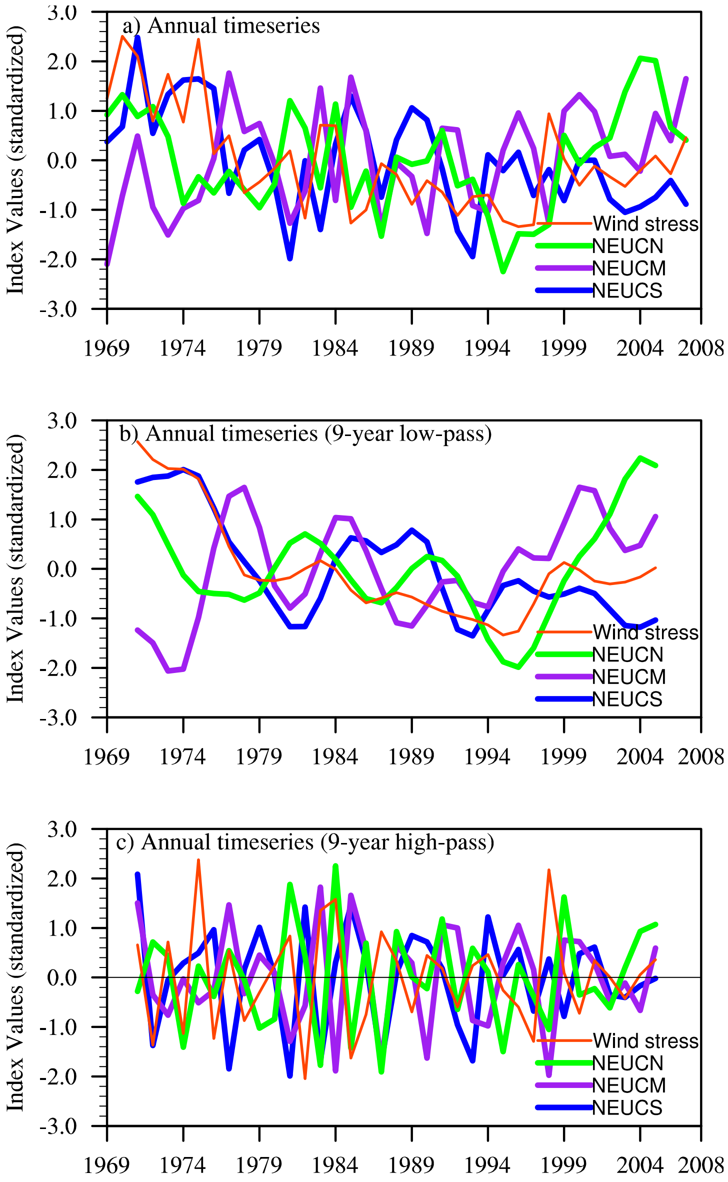

Figure S7. (a) The normalized annual mean indices for the NEUCS (blue), NEUCM (purple), and NEUCN (green) in cm/s, and the wind stress index (red). (b) and (c) are the same as (a), except for the 9-year low- and high-pass-filtered indices. The figure was made using NCL 6.4.0 (http://www.ncl.ucar.edu/).

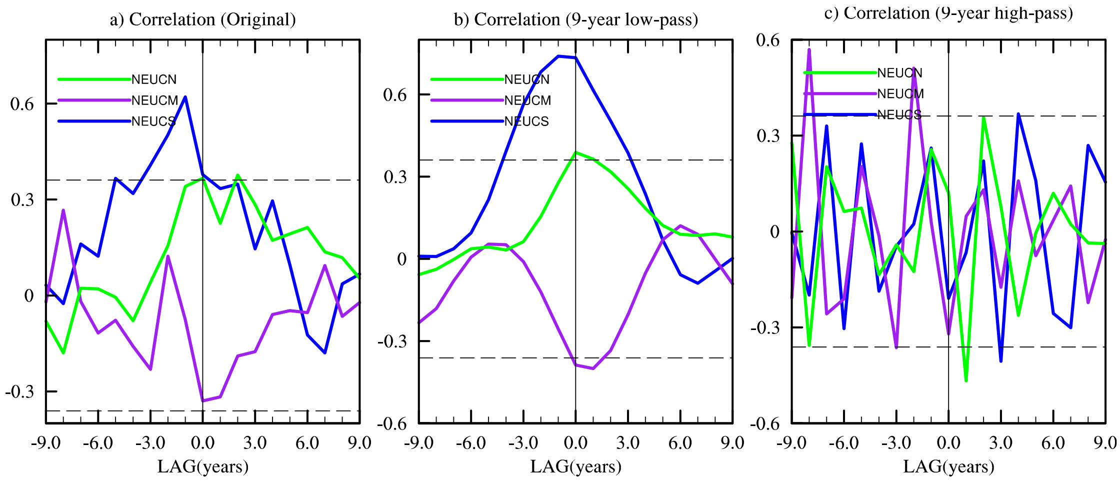

Figure S8. (a) The lead-lag correlation coefficients of wind stress index with the NEUCS (blue), NEUCM (purple), and NEUCN (green). (b) and (c) are the same as (a), except for the 9-year low- and high-pass-filtered indices. The dashed curves indicate the 95% significance level. The figure was made using NCL 6.4.0 (http://www.ncl.ucar.edu/).

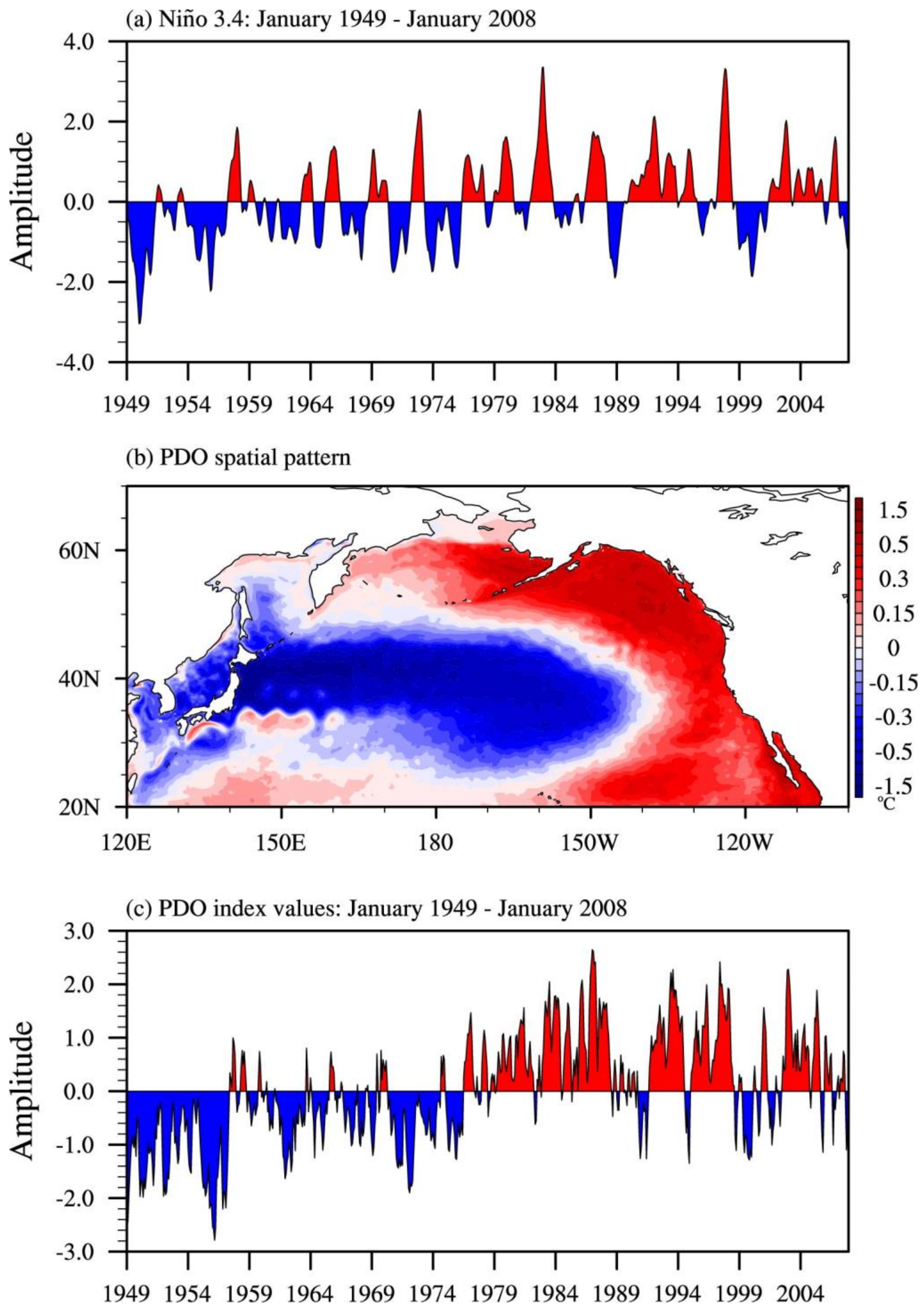

Figure S9. (a) 1949-2007 monthly mean series for sea surface temperature (SST) anomaly in Niño 3.4 region from LICOMH. (b) The leading empirical orthogonal function (EOF) pattern of monthly mean SST anomalies for the Pacific Ocean north of 20°N from LICOMH. (c) 1949-2007 monthly mean series for PDO index from LICOMH. The figure was made using NCL 6.4.0 (http://www.ncl.ucar.edu/).
